# Faculty perceptions of microlearning in health professions education: a mixed method analysis of implementation factors

**DOI:** 10.5116/ijme.68ff.c63e

**Published:** 2025-10-31

**Authors:** Nilesh Kumar Mitra, Norah Htet Htet, Vasudeva Rao Avupati, Fabian Davamani, Pamela David, Ker Woon Choy, Vishna Devi Nadarajah

**Affiliations:** 1 School of Medicine, IMU University, Bukit Jalil 57000, Kuala Lumpur, Malaysia; 2 School of Pharmacy, IMU University, Bukit Jalil 57000, Kuala Lumpur, Malaysia; 3 School of Health Sciences, IMU University, Bukit Jalil 57000, Kuala Lumpur, Malaysia; 4 Faculty of Medicine, Universiti Malaya, 50603 Kuala Lumpur, Malaysia; 5 Faculty of Medicine, Universiti Teknologi MARA, Sungai Buloh, 47000 Selangor, Malaysia; 6 Newcastle University Medicine Malaysia, Educity, 79200 Iskandar Puteri, Johor, Malaysia

**Keywords:** Microlearning, health professions education, faculty perceptions, implementation factors

## Abstract

**Objectives:**

This study aims to explore the preparedness of faculty in health professions education at three Malaysian universities by assessing their perceptions of basic concepts in microlearning as well as factors affecting effective content construction and digital format preferences.

**Methods:**

An explanatory sequential mixed-method approach was used to systematically analyse faculty perceptions by integrating quantitative and qualitative findings. A total of 121 faculty members voluntarily completed the online survey. A qualitative exploratory study was conducted with 20 selected staff members, followed by a thematic analysis. Descriptive and analytical statistics, including Pearson’s chi-square test, were used to analyse the data.

**Results:**

The survey revealed that 95.9% (n=116) of faculty members agreed that microlearning is ideal for the acquisition of microcontent with single learning outcomes. The optimal duration should be between 3 and 5 minutes. Strong associations [χ^2^(16, N=121) =33.17, p=0.007] between time duration and content size and content size and form of knowledge [χ^2^(16, N=121) =28.79, p=0.025] were observed in chi-square goodness-of-fit test. Microcontent of a single learning outcome, chunking of content, cognitive load, and degree to which topic connects with the media used emerged as primary sub-themes. Challenges in adapting skills to construct engaging microlearning content were highlighted.

**Conclusions:**

The study provides a microlearning framework for health professional educators to consider the complexity of content, its format, and integration with suitable digital tools. Future research should explore how combinations of microlearning and other instructional formats optimise learning outcomes.

## Introduction

The COVID-19 pandemic highlighted significant challenges in knowledge acquisition within educational institutions, prompting the adoption of flexible learning strategies.^1^ Microlearning has been defined as an instructional unit that provides a short engagement intentionally designed to achieve a specific, measurable outcome from the participant. An instructional unit can involve a learning activity, a video, a text message, work instructions, or a flashcard.^2^ Microlearning enhances learning efficiency by addressing reduced cognitive spans and providing learner’s significant control over their learning pace.^3^ The cognitive theory underlying microlearning, particularly the concept of the “forgetting curve,” suggests that individuals naturally lose information over time without reinforcement. This renders the short, focused nature of microlearning especially effective for knowledge retention.^4^ The concept of microlearning is grounded in cognitive load theory, which recognises the limitations of human working memory. Previous research has demonstrated that cognitive overload occurs when learners attempt to process excessive information simultaneously, which hinders effective learning.^5^ Microlearning addresses this challenge by breaking down complex subjects into manageable units, enabling learners to process information efficiently.^6^^,^^7^ Although microlearning has existed informally for years, its formalisation as a specific teaching strategy is relatively recent.^8^  Hug conceived microlearning as short, focused learning activities centred on small content units delivered over extended periods.^9^ This framework encompasses seven dimensions: learning time, content, curriculum, format, process connectivity, pedagogical approach, and learning media. The versatility of this framework has enabled microlearning to adapt across various disciplines, from business management to healthcare education. However, approaches to implementation varied significantly.^9^^, ^^10^^,^^11^ A review of the literature regarding microlearning supports the achievement of learning outcomes, learner engagement, knowledge retention, learner satisfaction, and feedback on application as evaluation metrics for microlearning content.^9^^,^^12^^-^^14^ Mobile devices, learning management systems, and multimedia platforms enable the creation and distribution of microlearning content with ease.^15^ This accessibility has contributed to the increasing popularity of microlearning, particularly among self-directed learners and professionals who balance education with other responsibilities and seek just-in-time knowledge. Microlearning supports educational equity by improving flexibility, personalisation and inclusivity.^16^ Despite extensive literature on microlearning in business and e-learning contexts, the factors regulating this educational environment remain predominantly descriptive and lack empirical support. An analysis of 476 publications from 2006 to 2019 revealed that 41% of microlearning research appeared as conference proceedings rather than as peer-reviewed articles.^17^ This indicates that cross-disciplinary and multi-institutional research studies can establish a comprehensive framework for the effective application of microlearning in higher education settings.

The healthcare domain presents unique considerations for the implementation of microlearning. Several successful implementations of microlearning in health professions education have been documented. Live video streaming of gynaecological surgeries has enabled easy delivery and access to microlearning content,^18^ while recording bedside nursing practices has demonstrated effectiveness among healthcare professionals.^19^ Just-in-time learning before critical procedures reinforces patient safety protocols.^20^ Despite the popularity of microlearning, pedagogical discomfort, technology inequalities, and privacy concerns have been identified in a scoping review.^21^ Additionally, instructor readiness and understanding of design and delivery principles represent significant implementation barriers. Medical education has traditionally relied on a comprehensive understanding of complex interconnected systems, raising questions about microlearning’s suitability for specific topics. Traditional teaching methodologies which adhere to structured curriculum formats often impede educators from exploring innovative teaching and learning approaches.^22^ The pandemic-induced shift from face-to-face instruction to online delivery prompted academics to develop microlearning content, despite the identified gaps in online learning resources and faculty readiness. Several studies have highlighted the gaps in online learning due to the ineffectiveness of the resources.^23^^-^^25^ Institutional support for microlearning implementation varies considerably across educational settings. Faculty development programs focusing on digital content creation often remain inconsistent, creating disparities in technological proficiency among educators. Furthermore, the time investment required to develop high-quality microlearning materials may not be adequately recognised in academic workload models, potentially discouraging adoption despite acknowledged pedagogical benefits.^26^

This study aims to explore faculty readiness for the implementation of microlearning by assessing their knowledge of basic concepts, the factors affecting effective content construction, and their perceptions regarding digital format preferences. By examining these elements through both quantitative and qualitative methodologies, this research aims to suggest a framework for integrating microlearning in health professions education.

## Methods

### Study context

This cross-sectional study engaged academic staff from medical and health sciences faculties at three universities during the 2023-2024 academic year. The study involved faculty from IMU University (Schools of Medicine, Health Science and Pharmacy), Universiti Malaya (Faculty of Medicine), and Universiti Teknologi MARA (Faculty of Medicine). The inclusion of multiple institutions enhanced the study’s representativeness and allowed for comparative analysis across different educational environments. IMU University employs a Key Clinical Problem (KCP)-based approach within body-system-based semesters, while the other participating institutions primarily utilise body-system-based modules. The three Universities represent a mix of public and private institutions with varying resource allocations for educational technology. The incorporation of various health science disciplines enabled the exploration of subject-specific factors that could influence the suitability of microlearning across diverse content areas.

### Study design

This study employed an explanatory sequential mixed-methods approach to systematically understand faculty perceptions by integrating quantitative and qualitative findings. In the first phase, we gathered faculty perceptions of microlearning through a questionnaire-based survey. After analysing the quantitative data, we examined the faculty’s understanding of constructing microlearning tools and the associated factors to clarify the findings of the quantitative study. As the study aimed to explore faculty readiness for microlearning, purposive sampling was used to include only academic staff who were familiar with the microlearning content. The target sample size was 250, with an anticipated 50 staff members from each of the five faculties. Participation was limited to pre-clinical academic staff without stratification by gender, age, or teaching subjects. The primary research instrument was a questionnaire previously used in 2012 for research on microlearning as a knowledge strategy process,^27^ and formal permission was obtained from the original source authors. The instrument was modified and tested with 30 academic staff members who had participated in a microlearning workshop. The reliability coefficient (Cronbach’s alpha) was 0.833 for the 28-item questionnaire, which improved to 0.847 after standardising several items. Ambiguous questions were removed, resulting in a 20-item questionnaire structured into three sections: basic concepts, factors affecting microlearning tool construction, and digital format preferences.

Ethical approval was obtained from the IMU University Joint Committee on Research and Ethics (IRB No. IMU 575-2023). The study adhered to principles of informed consent, confidentiality, and data protection. All data were anonymised during analysis and reporting to protect participant privacy. Faculty members received email invitations containing brief information about the research project, its objectives, the ethical approval process, and a link to an online questionnaire with informed consent provisions. The final sample consisted of 121 academic staff members across the three universities, representing a 48.4% response rate based on the initial target. The specific distribution of respondents across institutions included 52 participants from IMU University (27 from School of Medicine, 14 from School of Health Sciences, and 11 from School of Pharmacy), 38 from Universiti Malaya, Faculty of Medicine, and 31 from Universiti Teknologi MARA, Faculty of Medicine.

An explanatory sequential mixed-methods approach was planned to gather narrative data that could explain the observed numerical data from the survey. Qualitative data were collected through semi-structured interviews conducted via Microsoft Teams. The design of this qualitative exploratory interview study is based on the framework proposed by Maxwell 2008.^28^ Seven focus group discussions (FGDs) involved 20 faculty members from various disciplines, including Anatomy, Pathology, Biochemistry, Biology, Chemistry, English, Statistics, Immunology, Pharmacology, Clinical Skills, Cell Biology, and Medical Education. Each focus group consisted of 2-4 participants, allowing for in-depth discussion while ensuring diverse viewpoints. Interview guides were developed based on preliminary questionnaire findings, focusing on the explanations for the responses generated during the initial survey to clarify the quantitative study’s findings regarding the factors involved in creating microlearning content, including the role of digital formats. Each 45-minute interview was video-recorded and automatically transcribed using NVivo 14 software.

### Data analysis

Quantitative data were analysed using SPSS statistical software version 25 (IBM), employing descriptive statistics. Descriptive analyses included frequency distributions, percentages, median of Likert scale (1-5) and binary response patterns across questionnaire items. Box plots were created to visualise the distribution of the factors identified through the analysis of the faculty’s self-perceptions during the survey. The Pearson Chi-Square goodness-of-fit test was conducted to identify a significant association between the factors observed during the data analysis. For qualitative data, thematic analysis followed the approach of Braun and Clarke (2006),^29^ which involved a narrative review of interview transcripts to identify common themes. The analysis process consisted of six phases: familiarisation with data, generation of initial codes, theme searching, theme review, theme definition and naming, and report production. This structured approach ensured methodological rigour while allowing flexibility to capture emergent patterns in faculty perspectives. Although NVivo 14 generated initial codes, researchers manually reviewed transcripts to identify relevant codes and patterns, ultimately determining themes and sub-themes through collaborative deliberation and consensus. The two primary researchers independently coded a subset of transcripts before comparing coding schemes and resolving discrepancies through discussion. This process enhanced coding reliability and analytical depth. Questionnaire data provided breadth of coverage across a larger sample, while interview findings offered depth of understanding regarding faculty reasoning and contextual factors influencing perceptions.

## Results

### Basic concepts of microlearning

Most respondents (95.9%, n=116) agreed that microlearning aids in acquiring microcontent focused on single learning outcomes, while only 4.1% (n=5) disagreed. However, opinions diverged regarding completing multiple learning outcomes within a five-minute timeframe, with 52.1% (n=63) agreeing and 47.9% (n=58) disagreeing. This split opinion reflects the ongoing debate about the appropriate scope of content for microlearning units. A substantial majority (86%, n=104) believed microlearning effectively meets work-based knowledge needs, and nearly all participants (97.5%, n=118) agreed that microlearning applications evolve to meet the learners’ needs and technological advancements. Faculty often highlighted the advantages of microlearning for acquiring clinical skills and procedural knowledge during FGD, as it delivers concise, targeted instruction when needed, surpassing traditional educational methods. An overwhelming 99.2% (n=120) concurred that microlearning integrates well with personalised learning approaches, while 90.9% (n=110) felt microlearning could teach various subjects within medical and health science curricula. Several interviewees highlighted the potential for microlearning to support adaptive learning systems.

### Factors affecting the construction of microlearning tool

**Figure1:**
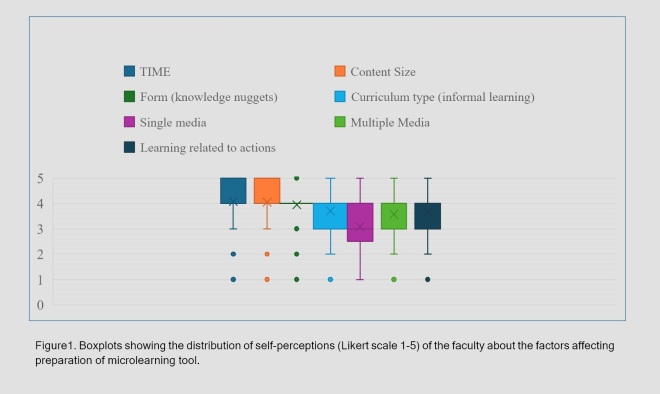
Boxplots showing the distribution of self-perceptions (Likert scale 1-5) of the faculty about the factors affecting preparation of microlearning tool.

The quantitative study highlighted time (duration), content size, form (knowledge nuggets), curriculum type, learning related to actions and media (single and multiple) as predominant factors affecting the preparation of the microlearning tool (Figure 1). The higher median of time (duration) and content size indicated that most respondents believed these two factors would play a principal role in the preparation of a microlearning tool. With a less varied distribution of perceptions, the form of the topic (knowledge nuggets) would also play a significant role. The distribution of perceptions regarding informal curriculum, media type, and learning related to actions indicated that these factors would play a less significant role in the preparation of a microlearning tool.

The association between time (duration) and content size [χ2(16, N=121) =33.17, p=0.007], as well as time (duration) and form of knowledge [χ2(16, N=121) =28.79, p=0.025], was shown to be significant in the chi-square goodness-of-fit test. A well-defined and concise content, broken into knowledge nuggets can effectively create a microlearning tool within a limited timeframe of 5 to 10 minutes. While the use of multiple media maintained a strong association with content size, no significant association was observed between other factors involved in preparing a microlearning tool. Both Figure 1 and Table 1 showed that the respondents consistently agreed that the use of multiple media would be more helpful in preparing a microlearning tool compared to the use of single media.

A significant majority (90.9%, n=110) considered it essential that chosen media effectively convey the microlearning topic. During FGD, several faculty members described the role of student feedback in the development processes, which helped refine media selection based on student feedback and learning outcomes.

### Digital format preferences

A significant majority of faculty members showed a preference for shorter videos, specifically those lasting 4 to 5 minutes [94.2%, χ2(1, N=114)=94.62, p=0.0002], compared to their preference for longer videos, which lasted between 15 and 20 minutes [85.1%, χ2(1, N=103)=59.71, p=0.0001] in the chi-square goodness-of-fit test. Brief podcasts of 2-3 minutes received greater support [72.7%, χ2(1, N=88) =25, p=0.0005] than longer 5–6-minute recordings [59.5%, χ2(1, N=72) =4.37, p=0.037]. This consistent preference for shorter formats across various media types reinforces the core microlearning principle of delivering brief and focused content. During the FGD, faculty often highlighted that student engagement noticeably declines after 4-5 minutes, regardless of the content’s quality or relevance. Infographics [90.9%, χ2(1, N=110) =83.33, p=0.0006] were deemed more suitable as a microlearning tool compared to text-heavy formats like PDFs [44.6%, χ2(1, N=54) =1.39, p=0.237]. Short videos featuring single animations gained significant approval [88.4%, χ2(1, N=107) =71.47, p=0.0002] compared to videos with multiple animations [64.4%, χ2(1, N=78) =10.12, p=0.001]. Interviewees consistently highlighted production quality factors, including audio clarity, visual design, narrative coherence, and technical accessibility, as critical success factors regardless of specific format selection (Table 2).

### Qualitative themes

The thematic analysis identified five primary themes: microlearning content duration, learning outcomes, suitability of basic science subjects, role in personalised learning, and the roles of multimedia and technology. Further analysis classified theme 1 into two sub-themes, theme 2 into three sub-themes, theme 3 into two sub-themes, and theme 4 into two sub-themes and theme 5 into four sub-themes (Table 3). This organisation ensured clarity while maintaining the richness of faculty perspectives. Faculty feedback indicated that the ideal duration of a microlearning tool should be between 3 and 5 minutes, primarily to reduce cognitive load and enhance student engagement. Seven interviewees highlighted student engagement through the use of three-minute microlearning videos.

The association between the factors affecting the preparation of the microlearning tool observed in the quantitative study (Table 1) was explained by the analysis of the qualitative study (Table 4). A single learning outcome and reduced cognitive load, highlighted as a sub-theme in the qualitative study, were explained through corresponding quotes, which underscored the strong association between the time (duration) and content size. The chunking principle, explained by the analogy of small bites and the suitability of microcontent, proved the strong association between content size and the form of knowledge. A pharmacology lecturer presented the example of a clearly visible adverse drug reaction, such as skin rashes, which has become a popular microlearning content, to correlate the nature of the concept and how well the subject connects with the media used. During the FGDs, the suitability of basic science subjects as microlearning content was often discussed, and the discussion proved that even knowledge nuggets of a misfit curricular type would not make an engaging microlearning tool. Under the theme of the role of microlearning in personalised learning, skills related to work-based knowledge (such as wound dressing or hand washing techniques) were referred to by the interviewees, explaining the association between the form of knowledge and learning related to the actions.

**Table 1:** Summary of the chi-square test to show significant association between the factors affecting preparation of microlearning tool

Factor Pair	Chi-Square (df)	p-value^**^	Cramér’s V
1. Time vs Content Size^*^	33.17 (16)	0.007^**^	0.007
2. Time vs Form (knowledge nuggets)^ *^	28.79 (16)	0.025^**^	0.025
3. Content size vs Form (knowledge nuggets)^ *^	59.09 (16)	0.001^**^	0.001
4. Form (knowledge nuggets) vs Curriculum type^*^	14.97 (16)	0.526	0.526
5. Single media vs Multiple media^*^	23.97 (16)	0.094	0.094
6. Content size vs Multiple Media^*^	84.01 (16)	0.001^**^	0.001
7. Form (knowledge nuggets) vs learning related to actions^*^	23.62 (16)	0.098	0.098

* Likert scale (1-5)

**Statistically significant p<0.05

**Table 2:** Faculty self-perceptions on the digital format suitable for a microlearning tool (N=121)

Format	Agreen (%)	Disagreen (%)	Chi-Square(df)^*^	p-value^**^
1. Short video (4 to 5 minutes)	114 (94.21)	7 (5.79)	94.62 (1)	0.0002^**^
2. Long video (15 to 20 minutes)	103 (85.12)	18 (14.88)	59.71 (1)	0.0001^**^
3. Podcast (2 to 3 minutes)	88 (72.73)	33 (27.27)	25 (1)	0.0005^**^
4. Podcast (5 to 6 minutes)	72 (59.5)	49 (40.5)	4.37 (1)	0.037^**^
5. Short video with a single animation	107 (88.43)	14 (11.57)	71.47 (1)	0.0002^**^
6. Short video with multiple animations	78 (64.46)	43 (35.54)	10.12 (1)	0.001^**^
7. PDF to provide information	54 (44.63)	67 (55.37)	1.397 (1)	0.237
8. Infographics	110 (90.9)	11 (9.1)	83.33 (1)	0.0006^**^

*Chi-Square goodness to fit test

** p<0.05 with alpha level 0.05

## Discussion

### Basic concepts and factors affecting construction of microlearning tool

This study reveals generally positive perceptions toward microlearning among health professional faculty, indicated by a strong association between the factors for preparing microlearning content, including time duration and content size, as well as an association between content size and the form of knowledge in the form of nuggets. The generation of sub-themes, single learning outcome, reduced cognitive load, microcontent, chunking as an essential element as well as assessment of learning outcomes, highlighted the role of microlearning in facilitating microcontent acquisition for single learning outcomes. These findings align with broader educational trends that embrace focused, modular learning to accommodate contemporary learning preferences and cognitive constraints. The strong support from faculty indicates the potential for broader implementation of microlearning within the field of health professions education, specifically for discrete and well-defined learning objectives. Qualitative findings identified three key sub-themes related to learning outcomes: the microcontent of a single learning outcome, digestible micro-content chunks, and the integration of assessments within content, enabling students to take responsibility for their learning completion. This finding aligns with a study, which noted that microlearning modules typically focus on specific learning objectives with on-demand accessibility.^30^ The study by Rof and coleagues (2024) on learner satisfaction with microlearning formats supports our finding of high agreement on microlearning’s effectiveness for single learning outcomes.^31^

Several interviewees highlighted that certain subjects require critical thinking and in-depth knowledge, such as advanced mathematics or engineering, and those concepts are not suitable to be delivered through microlearning. Complex clinical topics may require longer durations, suggesting that microlearning might require supplementation with other educational strategies to achieve a comprehensive understanding of complex subjects.^32^ This tension between comprehensiveness and digestibility constitutes a fundamental challenge in the implementation of microlearning. Faculty members teaching systems-based subjects, such as physiology or integrated clinical reasoning, expressed greater reservations about incorporating meaningful content within brief timeframes. Conversely, those teaching discrete skills or factual knowledge reported success with even shorter durations. This disciplinary variation suggests the need for flexible implementation approaches rather than one-size-fits-all microlearning models. Two sub-themes emerged related to the theme, role of microlearning in personalised learning: work-based knowledge and matching skills with timing. Many of the existing microlearning videos focus on work-based knowledge, and outcomes related to this knowledge can be effectively completed using a microlearning tool. This perception is supported by research that highlights the importance of just-in-time learning, particularly for professionals needing quick access to relevant information during clinical practice.^17^ The thematic analysis confirmed that the ideal duration for a microlearning tool should be between three and five minutes. Arabi and colleagues reported an 18% increase in positive course reviews after converting longer videos into 3–5-minute segments.^33^

### Digital format preferences

The preference for multimedia platforms over single media indicates educators’ willingness to explore diverse media platforms. A quasi-experimental study found that 100% of students preferred videos designed according to multimedia design principles.^34^ This preference is consistent with cognitive load theory and multimedia learning principles, which indicate that suitable combinations of visual and auditory information can improve learning by utilising diverse processing mechanisms channels. Actions can be effectively integrated into microlearning activities, which may foster reflective practice as learners recall and self-assess their actions. During the FGD, faculty teaching clinical skills described significant improvements in skill acquisition when procedural demonstrations were broken into discrete steps through microlearning modules, allowing focused mastery of each component. The high agreement on breaking topics into small segments and presenting manageable content nuggets aligns with microlearning’s core concept of small-step learning supported by small content or activity blocks.^35^ Short 4-5 minute videos were preferred over longer formats, consistent with research showing increased student engagement and knowledge retention with brief videos.^36^ The faculty reported higher completion rates and better assessment outcomes with shorter videos than longer ones on the same content, indicating real pedagogical benefits beyond just shorter attention spans.

### Implementation challenges

The faculty identified several implementation challenges, including ensuring microlearning does not oversimplify complex academic content. For subjects requiring deep understanding and critical thinking, microlearning should complement rather than replace traditional instructional methods. This balanced perspective acknowledges microlearning’s value while recognising its limitations, suggesting thoughtful integration rather than wholesale replacement of existing educational approaches. Additionally, faculty adaptation to creating content with complex multimedia types should be considered when implementing animation or podcast-based materials. Technical barriers varied considerably based on age, experience, institutional support, and individual interest. While some institutions provided comprehensive training and production assistance, others left faculty largely self-sufficient in developing technical skills. This variation in support created implementation disparities that influenced both faculty willingness to create microlearning content and the quality of resulting materials.

**Table 3:** Final themes and sub-themes of qualitative analysis

Themes	Sub-themes
Theme 1	
Duration of microlearning content.	Number of learning outcomes.
	Cognitive load.
Theme 2	
Learning outcomes of microlearning content.	Microcontent of a single learning outcome.
	Chunking of the content as an essential element.
	Assessment of learning outcome.
Theme 3	
Suitability of basic science subjects as microlearning content.	Nature of the concept.
	How well the subject connects with the media used.
Theme 4.	
Role of microlearning in personalized learning.	Work-based knowledge.
	Matching skills with the timing.
Theme 5	
Role of multimedia and technology.	Engagement with the students.
	Alignment of the media with the format of the content.
	Ease of production of the video.
	Adaptation of the skills by the lecturers.

**Table 4:** Integrated results matrix illustrating the relationship between the factors affecting the preparation of the microlearning tool and the qualitative results

Quantitative results	Qualitative results	Example quote
Significant association between Time and Content Size.	“Number of learning outcomes” and “Cognitive load” as sub-themes under the theme of “Duration of microlearning content.”	“Because this is Microlearning, the ideal is to get away from the cognitive overload. So, my perspective is that there should be one learning outcome.” (Interviewee 01)“You get 5 minutes to 10 minutes and one learning outcome, and then you get 15 minutes with three learning outcomes, I will take only one learning outcome, less than 10 minutes.” (interviewee 05)
Significant association between Content size and Form (knowledge nuggets).	“Microcontent of a single learning outcome” and “Chunking of the content as an essential element” as sub-themes under the theme of “Learning outcome.” Learning outcomes are already an essential sub-theme of the duration.	“In a very simple context, when you are eating, it is easier if you eat in small bites; you can chew it well. You can enjoy the food.” (Interviewee 17)“Oh, not all subjects. Certain subjects require critical thinking and in-depth knowledge, like advanced maths or certain forms of engineering. So, in those difficult areas, we cannot use microlearning.” (Interviewee 04)
No significant association between Form (knowledge nuggets) and Curricular type.	“Nature of the concept” and “How well the subject connects with the media used” as sub-themes under the theme of “Suitability of basic science subjects as microlearning content.”	“So maybe if you’re teaching adverse drug reaction, something is there which is very visible you can see in day-to-day use, you can use it in your video and then naturally student will be engaged and it will be in their impression that adverse reaction, once they see the video they will not forget. But not always the learning component, we decide, can get the visible example.” (Interviewee 09)“For example, like neuroanatomy, it just cannot be covered in that 10 minutes. You need to have a wider understanding.” (Interviewee 17)
Significant association between Content size and Multiple media	“Engagement with the students,” “Alignment of the media with the format of the content” and “Adaptation of the skills by the lecturers” as sub-themes under the theme of “Role of multimedia and technology”	“Format of presentation, multimedia tools and handheld devices, if they are attractive and can engage the students, then microlearning can meet the outcomes.” (Interviewee 13)
Significant association between Form (knowledge nuggets) and Learning related to actions	“Work-based knowledge” and “Matching skills with the timing” as sub-themes under the theme “Role of microlearning in personalized learning.”	“I’m a fresh graduate, running OPD. If a diabetic foot patient comes requiring dressing, I will quickly go to YouTube, find out the diabetic foot dressing technique and implement it on the patient so that it is just like 1 objective.” (Interviewee 19)

### Limitations

This study has several limitations. As a cross-sectional design conducted among academic staff from three institutions, the findings reflect perceptions at a single point in time and may not capture changes in attitudes or readiness over longer periods. The focus on pre-clinical faculty, while novel for exploring microlearning, limits generalisability across the broader academic workforce in medical programmes. Although including multiple institutions improved representativeness, reliance on purposive sampling and a response rate of 121 faculty members may have caused selection bias, where those interested in microlearning but unfamiliar with the tool were more likely to participate. Furthermore, as the study was conducted in three institutions within a single country, findings may reflect country-specific educational policies, institutional cultures, and resource contexts, which limit their transferability to other regions or health systems.

## Conclusion

This mixed-methods study examined medical and health sciences faculty perceptions regarding microlearning across basic concepts, construction factors, and digital format preferences. The findings from three institutions provided valuable insights across diverse preclinical disciplines and educational environments. This strengthens and broadens the applicability for the implementation of microlearning in health professions education. Faculty perceptions toward microlearning were largely positive, with several contributing factors: micro-content learning outcomes, manageable learning components, adaptability to learner needs, work-based learning compatibility, short 3–5-minute durations, and personalised learning alignment. Interestingly, quantitative analysis identified short duration and content size as key construction factors, while qualitative analysis highlighted the microcontent of single learning outcomes, chunking of content, and the nature of content, along with suitability for the media. Although quantitative findings suggested faculty confidence in teaching various subjects through microlearning, focus group discussions revealed challenges with complex topics. The mixed responses regarding the sufficiency of microlearning for complex outcomes suggest its optimal use as a complementary strategy alongside traditional teaching methods. Future research should explore how different combinations of microlearning and other instructional formats optimise learning outcomes across various medical disciplines and during clinical training. The study highlighted preferences for short videos, infographics, and animations to satisfy student desires for concise, visually appealing content. The criteria identified provide a framework for educators to evaluate and select appropriate modalities before constructing microlearning tools. Future research should investigate how these criteria impact learning outcomes across various educational settings and learner demographics. Ultimately, microlearning represents a valuable addition to health professions education when thoughtfully implemented with consideration for content complexity, format appropriateness, and integration with existing pedagogical approaches.

### Acknowledgments

The authors acknowledge the assistance of Shamala Ramasamy in statistical analysis. The authors also acknowledge the assistance of faculty members from IMU University, Universiti Malaya and Universiti Teknologi Mara who participated in the study.

Funding: This work was supported by the IMU University Joint Committee on Research and Ethics. Grant IRB No. IMU 575-2023.

### Conflict of Interest

The author**s** declare that there is no conflict of interest.
